# Genomic adaptation of admixed dairy cattle in East Africa

**DOI:** 10.3389/fgene.2014.00443

**Published:** 2014-12-19

**Authors:** Eui-Soo Kim, Max F. Rothschild

**Affiliations:** Department of Animal Science, Iowa State UniversityAmes, IA, USA

**Keywords:** admixture mapping, selection signatures, adaptation to new environment, population structure, gene flow, crossbred cattle

## Abstract

Dairy cattle in East Africa imported from the U.S. and Europe have been adapted to new environments. In small local farms, cattle have generally been maintained by crossbreeding that could increase survivability under a severe environment. Eventually, genomic ancestry of a specific breed will be nearly fixed in genomic regions of local breeds or crossbreds when it is advantageous for survival or production in harsh environments. To examine this situation, 25 Friesians and 162 local cattle produced by crossbreeding of dairy breeds in Kenya were sampled and genotyped using 50K SNPs. Using principal component analysis (PCA), the admixed local cattle were found to consist of several imported breeds, including Guernsey, Norwegian Red, and Holstein. To infer the influence of parental breeds on genomic regions, local ancestry mapping was performed based on the similarity of haplotypes. As a consequence, it appears that no genomic region has been under the complete influence of a specific parental breed. Nonetheless, the ancestry of Holstein-Friesians was substantial in most genomic regions (>80%). Furthermore, we examined the frequency of the most common haplotypes from parental breeds that have changed substantially in Kenyan crossbreds during admixture. The frequency of these haplotypes from parental breeds, which were likely to be selected in temperate regions, has deviated considerably from expected frequency in 11 genomic regions. Additionally, extended haplotype homozygosity (EHH) based methods were applied to identify the regions responding to recent selection in crossbreds, called candidate regions, resulting in seven regions that appeared to be affected by Holstein-Friesians. However, some signatures of selection were less dependent on Holsteins-Friesians, suggesting evidence of adaptation in East Africa. The analysis of local ancestry is a useful approach to understand the detailed genomic structure and may reveal regions of the genome required for specialized adaptation when combined with methods for searching for the recent changes of haplotype frequency in an admixed population.

## Introduction

Dairy cattle have been successfully improved to optimize their performance under favorable environments. During the recent decades, Western or exotic dairy cattle have been imported rapidly to diverse geographic regions using new technologies such as artificial insemination (AI). However, purebreds have rarely adapted well to new environments in a short period of time and usually not without significant interventions for health. Holstein-Friesians, considered by many to be the most productive dairy breed in temperate regions, have been imported to tropical or subtropical regions in an attempt to improve milk production, but in many cases they have shown poor performance in comparison with that obtained in the temperate climate (Bohmanova et al., 2007).

In East Africa, European cattle have been introduced since the early twentieth century, creating local cattle herds under strong natural selection by disease and environmental factors. According to a previous study, mostly European ancestry remains in a large herd Kenyan cattle population sample but with little native germplasm (Gorbach et al., 2010) and the Kenyan cattle that have been maintained in local smaller farms were mostly an admixed population of imported breeds, including Holstein-Friesian and Guernsey. Genetic variation between breeds for most quantitative traits represents opportunities to combine breeds to improve productivity (Van Vleck et al., 1986). Despite a presumed advantage of crossbreeding in the F1 generation profit from crosses between Holsteins and other dairy breeds in the United States is not as high as that of Holsteins (VanRaden and Sanders, 2003). Nevertheless, mating between cattle breeds with diverse genetic backgrounds that may increase the adaptability of cattle in other geographic regions.

In Africa, crossbreds resulting from a limited gene pool of exotic cattle imported from more productive countries and strong natural selection for a resistance to infectious disease or other environmental stresses possibly may display a footprint of selection. In particular, animals bred over time in local farms are more likely to be adapted to a new environment, allowing us to obtain clues to reveal the genomic region involved in adaptation. To identify genomic regions corresponding to natural selection, new approaches have been suggested using large scale genetic markers (Nielsen et al., 2007). In Holstein cattle, signatures of artificial selection have been identified by examining the decay of extended haplotype homozygosity (EHH) (Qanbari et al., 2010; Elferink et al., 2012; Glick et al., 2012) or F_ST_ (Barendse et al., 2009; Flori et al., 2009) with other cattle breeds. Common signatures of selection have been reported in Holstein-Friesians and those are expected to be detected in addition to the unique signatures of selection in cattle sampled from East Africa. Moreover, admixture mapping (Winkler et al., 2010) was applied to infer the background of genomic regions considering the admixed population structure in Kenyan cattle. Since animals in local farms have been maintained by crossbreeding with mostly European or U.S. dairy cattle breeds, inferring local ancestry would help to reveal the genetic background contributing to adaptation in East Africa. This approach could be an attractive complementary analysis to signatures of selection for the analysis of an admixed population. Admixture mapping may also help to reveal favorable parental populations in a genomic region as a result of adaptation.

Large variation in production ability of exotic cattle has been reported in non-temperate climate regions (O'Neil et al., 2010). There is an opportunity to improve livestock when the genes involved in desirable phenotypes are identified in a specific environment. In developing countries, sparse recording of livestock performance and limited use of such records have been obstacles to improve the genetic ability of animals. Additionally, economic traits, including resistance to infectious disease, improved fertility, heat tolerance, or more basic information have not been recorded in many regions due to the limited availability of time, and effort and overall cost. Thus, identification of genomic regions and genes adapted to a specific condition may help breeders to understand the genetic characteristics of population and to establish a plan to improve cattle under severe conditions.

## Materials and methods

### Genotypes and population

Based on a previous study, we consider the following animals a crossbred group: 20 bulls that were have been heavily used in breeding programs for genetic improvement of the dairy cattle population in Kenya, and 142 cows and offspring sampled from many small farms in Kenya (crossbreds). Phenotypes of individual bulls and cows were not available. There were 25 animals across four generations from a large Kenyan ranch that were known to be closely related to Holsteins and Friesians (Friesians). DNA was extracted from blood samples and genotyped using the Illumina 50K bovine SNP chip (San Diego, CA, USA). Markers with minor allele frequency >0.01 within any breed were included in the analysis. We used Plink (Purcell et al., 2007) to control the quality of data and the position of each SNP on a bovine chromosome (BTA) was decided using genome assembly UMD 3.1. In order to assess the genetic background of Kenyan cattle, SNP genotypes of Holstein (*n* = 62), Guernsey (*n* = 21), and Norwegian Red (*n* = 21) cattle were obtained from the HapMap project (Bovine HapMap Consortium, 2009). Norwegian Red cattle share the many common ancestors with Ayrshire that have been imported to Kenya.

### Population admixture

Principal component analysis (PCA) and discriminant analysis of principal components (DAPC) were used to examine the admixture in the Kenyan cattle population. DAPC is an approach that optimizes the separation of individuals into predefined groups using a discriminant function of principal component (Jombart et al., 2010). Based on DAPC, membership probability was obtained to present the overall genetic background of an individual. For this analysis, Western breeds, namely Holstein, Guernsey, and Norwegian Red cattle as well as two Kenyan cattle populations were included. Differentiation of populations was also measured by estimation of single marker F_ST_ (Wright, 1951) across the genome. The software package *adegenet* in R was used to analyze population admixture and F_ST_ (Jombart and Ahmed, 2011).

### Inference of local ancestry

Although ancestries in the Kenyan dairy cattle of admixed individuals appear complicated, the majority of their ancestors were known to originate from a limited number of imported breeds, particularly Holstein-Friesian and Guernsey (Gorbach et al., 2010). To further understand the observed admixture, we estimated local ancestries at each locus in Kenyan crossbred cattle comparing with animals sampled from their parental breeds. The SNP allele frequency of haplotypes obtained from three European/U.S. dairy breeds was applied to infer the ratio of the parental breeds in a specific genomic regions using LAMP-LD (Baran et al., 2012). This method applied hidden Markov models (HMM) to trace the origins of an admixed population based on the haplotype patterns trained using parental breeds. The genome was divided into non-overlapping 30-SNP window to train the HMM of the ancestral population. The LAMP-LD classifies the origin of a haplotype in crossbreds into at most two ancestral groups. Thus, we could obtain the mean and minimum probability of local ancestry. When one haplotype originated from only one parental breed, it was regarded as true local ancestry to calculate minimum probability, while multiple ancestries were allowed to calculate mean probability of local ancestry. For local ancestry mapping, haplotype phase was decided using Beagle package (Browning and Browning, 2007).

### Migration of haplotypes from parental breeds to Kenyan crossbreds

In addition to local admixture analysis, frequencies of haplotypes from animals in parental breed were examined in crossbred cattle. We suggest this method to test whether the frequency of a haplotype from Holstein, Ayrshire, or Guernsey cattle has changed substantially in Kenyan crossbreds compared to the frequency of the haplotype in a parental breed. The most frequent haplotypes, probably a signature of the influential bulls, were compared between animals in a breed and crossbreds to assess the migration of haplotypes under selection using a 30-SNP window and starting with a new window every 15-SNPs. Mean and standard deviation of the length of 30-SNP haplotype was 1.48 and 0.38 (Mb), respectively. Next, the difference in the frequency of a common haplotype which originated from a parental breed was calculated using the equation, *freq*(*D*) = *p*(*B*) × *freq*(*K*|*B*) − *freq*(*B*), where *freq*(*B*) is the frequency of the most common haplotype of a breed, *p*(*B*) is the ratio of an ancestry in the region encompassing the haplotype, and *freq*(*K*|*B*) is the frequency of haplotype that originates from a parental breed in Kenyan crossbreds. In order to calculate the expected frequency of the haplotype from a parental breed in crossbreds, the ratio of local ancestry, *p*(*B*), was obtained from the results of LAMP-LD. Then, a standardized score was computed by $\frac{\left\{freq(D)-mean(freq(D))\right\}}{stdev(freq(D))}$, where *mean*(*freq*(*D*)) and *stdev*(*freq*(*D*)) are the mean and standard deviation of *freq*(*D*), respectively. This score accounts for a migration of the most frequent haplotype from a parental breed to Kenyan crossbred cattle. To calculate scores, the most frequent haplotypes that were also found in Kenyan crossbreds were included. Perl and R scripts were used to assess the flow of haplotypes.

### Signatures of selection

The evidence for positive selection was determined by calculating the value of the standardized integrated EHH (iHS) that measures the relative decay of EHH of the ancestral and derived core allele (Voight et al., 2006). This test was applied to detect selection signatures in Kenyan crossbreds, and presumed parental breeds. Similarly, a comparative EHH score, Rsb (Tang et al., 2007), was calculated to compare the relative decay of EHH for each marker between populations. Using Rsb, we compared the EHH of Kenyan bulls derived from Holstein-Friesians and Holsteins in the United States. The *rehh* R package was used to compute the values of iHS and Rsb with default parameters (Gautier and Vitalis, 2012).

### Annotation

Genes in the regions determined by local ancestry mapping and selection signatures were considered to be from candidate regions and retrieved from Biomart in Ensembl (EMBL-EBI). Using Enrichr (Chen et al., 2013) and WikiPathways (Kelder et al., 2009), biological function of genes was annotated.

## Results

### Admixture in Kenyan cattle and imported breeds

**Figure 1:**
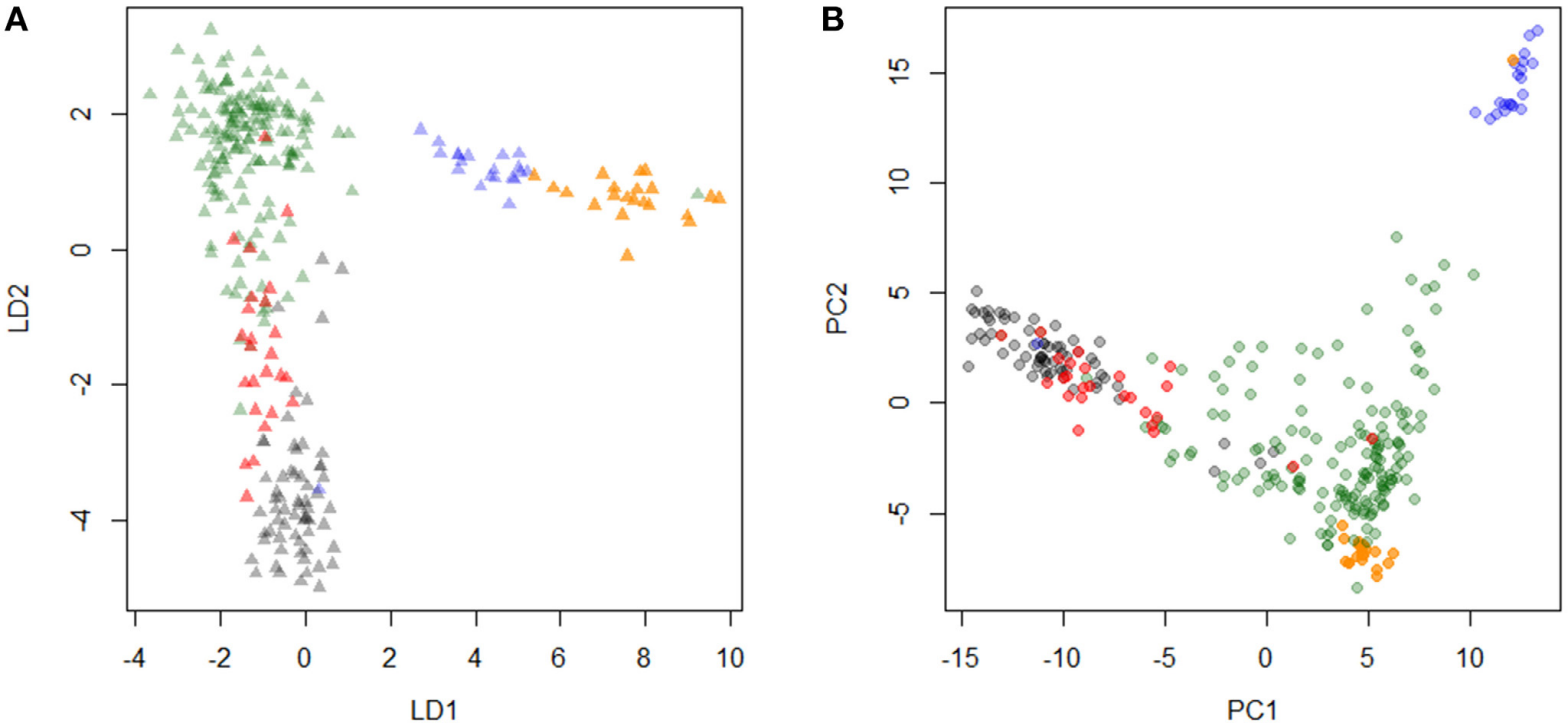
**Principal component analysis of Kenyan and other dairy cattle breeds**. Discriminant analysis of principal component (DAPC) and principal component analysis (PCA) are plotted (**A,B**, respectively). Each color represents black, Holstein; red, Kenyan Friesian; green, Kenyan crossbreds; blue, Guernsey; orange, Norwegian Red.

The PCA and DAPC provided evidence that Friesian type cattle from the large ranch were highly correlated with Holstein cattle, whereas the animals sampled from smaller farms were admixed populations of Holstein-Friesian, Norwegian Red (or Ayrshire), and Guernsey cattle. Using DAPC with five predefined breeds, crossbreds appeared as an admixed population with Holstein and Kenyan Friesian, but relatively unrelated to Guernsey or Norwegian Red (Figure 1A). Three breeds, including Holsteins, Kenyan Friesians, and Kenyan crossbreds were separated by the second linear determinant (LD2), and the first linear determinant (LD1) reflected the relatively lower influence of Guernsey and Norwegian Reds in crossbred cattle. PCA plots showed similar clustering of sampled breeds except Norwegian Red that were plotted in the middle of a broad cluster of crossbreds (Figure 1B), which was in an agreement with history of Holstein-Friesians in Kenya.

### Local ancestry in Kenyan crossbred cattle

**Figure 2:**
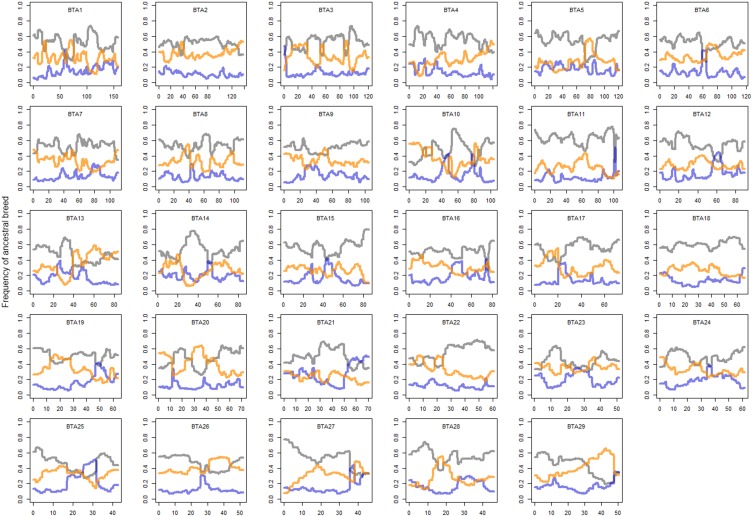
**Local ancestry of crossbreds**. Gray, orange, and blue lines represent Holstein, Norwegian Red, and Guernsey, respectively. Mean local ancestry of SNP is plotted on genomic position of each chromosome. y axis is percentage ancestral breed and x axis genomic position.

In order to clarify the genome-wide pattern of admixture, local ancestry of cattle was inferred using the haplotype information from dairy breeds related to Kenyan crossbreds. Overall, more than half of haplotypes were inferred to be shared with those of Holsteins (0.53 ± 0.097) under the assumption of admixture with three ancestral dairy cattle breeds (Holstein, Guernsey, and Norwegian Red). When comparing the other two breeds, the genetic background of Norwegian Red cattle (0.32 ± 0.096) was found to be more influential than the ancestry from Guernsey (0.15 ± 0.078) in the Kenyan admixed population. Local ancestry of most regions was not solely dominated by an ancestral breed, whereas the ancestry of Holstein-Friesian was substantial when the influence of a breed was relatively high (>0.75), particularly in regions on BTA 10, 14, 15, or 27 (Figure 2). The probability of ancestry was also calculated based on the haplotypes that originated exclusively from an ancestral breed (Figure S1). Interestingly, we found Norwegian Reds shared a considerable number of common ancestors with Holsteins, but the majority of haplotypes that derived from only Holstein was great compared to other breeds (Figure S1), representing the strong impact of Holstein-Friesians in Kenyan crossbreds. Nonetheless, selection signatures were not dependent on the ratio of Holstein background in crossbreds. When examining the local ancestry, we found that the ratio of Holstein background was relatively low (<0.5) in the regions on BTA 4 (40 Mb), 7 (45 Mb), 10 (85 Mb), 13 (45 Mb), 20 (20–40 Mb), and 26 (25 Mb).

### Haplotypes flow from Holsteins to Kenyan crossbreds

**Figure 3:**
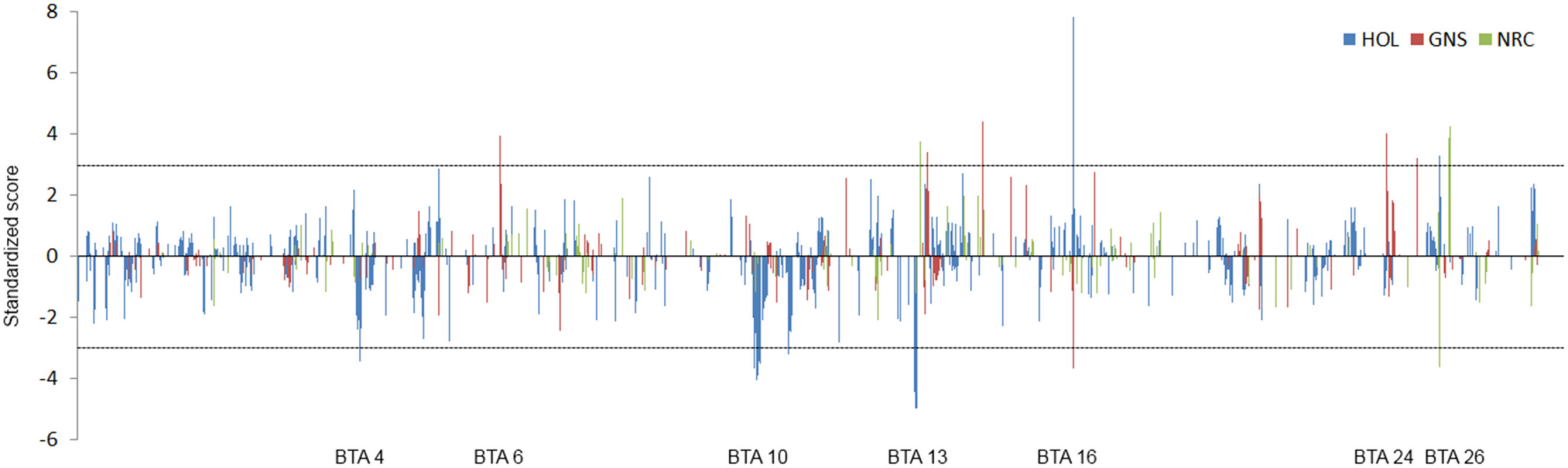
**Migration of the most frequent haplotypes from parental breeds to Kenyan crossbreds**. The standardized score of haplotype migration of Holstein (blue), Guernsey (red), and Norwegian Red (green) are shown across the genome. Positive score represents the higher frequency than expected frequency of the most frequent haplotype that is originated from Holsteins. Negative value represents the opposite case of positive score. Number on x axis shows chromosome.

**Figure 4:**
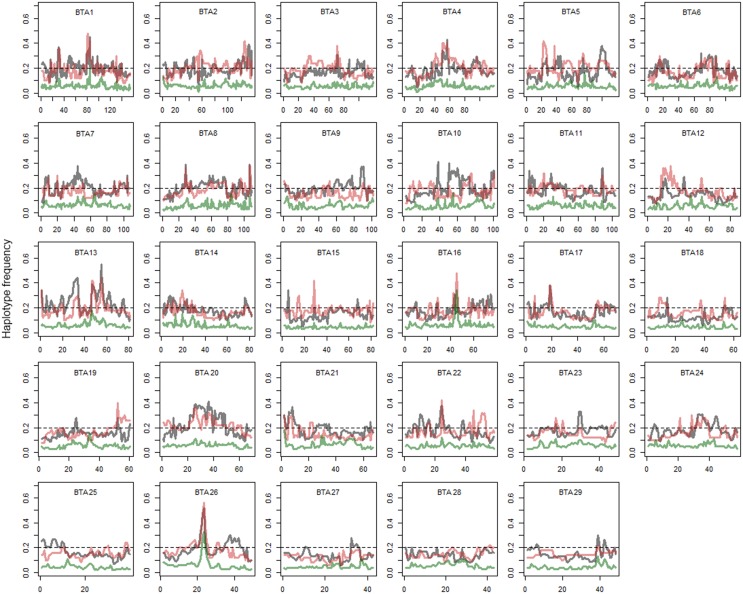
**The most frequent haplotypes in Holstein, Kenyan Friesian, and crossbreds**. Gray, red, and green lines represent Holstein, Kenyan Friesian, and Kenyan crossbreds, respectively. Gray line, Holstein; Red line, Kenyan Friesian; Green line, Kenyan Crossbred. Frequency of the most common haplotype in each breed is shown. y axis is frequency of most frequent haplotype and x axis shows genomic position (Mb).

The analysis of local admixture helps to infer the overall genetic background of cattle of the region. However, selection signatures are mainly detected by long haplotypes shared by individuals. To reveal the origin of haplotypes, we compared the most frequent haplotypes of crossbreds with their parental breeds (Figure 2; Figure S2). As shown above, a considerable amount of consensus haplotypes was found across the genome in all Kenyan dairy cattle (Figure 3), in particular those haplotypes with high frequency (>0.3). Approximately, 26% of the most frequent haplotypes originated from Holsteins were found in Kenyan crossbreds, whereas less than 10% of the most frequent haplotypes of Guernsey (8%) or Norwegian Red (6%) were identical to any haplotype in Kenyan crossbred cattle. In most genomic regions, frequency of the haplotype in Kenyan crossbreds was dependent on the frequency of haplotypes within their ancestral breed (Figure 3). Despite the fact that the most frequent haplotypes on BTA 10 and 13 remained, these appeared to be unfavorable haplotypes when considering the difference of the expected and observed frequencies in Kenyan crossbreds (Figure 3, Table 1). The frequency of the most common haplotype in crossbreds was ~0.3 at 25 Mb on BTA 26, which originated from Holstein-Friesians. In a region on BTA 16, the difference of haplotype frequency between Holsteins and crossbreds was the highest (0.07 vs. 0.34), whereas an identical haplotype was found in all imported breeds in the region from 23 to 24 Mb, resulting in the high levels of the common haplotypes in Kenyan crossbreds (Figure 4).

**Table 1:** **Comparisons of the most frequent consensus haplotype in parental breeds and Kenyan crossbreds**.

**Parental breed^a^**	**BTA**	**Region (Mb)**	**Frequency in parental breed^b^**	**Expected frequency^c^**	**Observed frequency^b^**	**Frequency change^d^**	**Standardized score^d^**
HOL	4	49.22–57.03	0.43	0.11	0.29	−0.18	−3.11
GNS	6	62.96–64.29	0.50	0.21	0.09	0.12	3.94
HOL	10	51.97–66.12	0.34	0.25	0.05	−0.20	−4.08
HOL	13	28.92–34.03	0.44	0.29	0.05	−0.24	−5.00
GNS	13	49.86–51.78	0.67	0.19	0.09	0.10	3.40
HOL	16	43.30–45.76	0.16	0.07	0.34	0.27	7.83
GNS	16	44.05–45.76	0.52	0.19	0.34	−0.15	−3.66
GNS	24	34.81–39.06	0.48	0.18	0.06	0.12	4.03
HOL	26	22.31–24.91	0.52	0.25	0.34	0.09	3.13
NRC	26	22.31–24.91	0.45	0.18	0.33	−0.15	−3.62
NRC	26	37.33–39.74	0.38	0.21	0.05	0.16	4.26

a *HOL, GNS, and NRC stand for Holstein, Guernsey, and Norwegian Red cattle, respectively*.

b *Haplotype frequency > 0.3 in a parental breed or Kenyan crossbreds is shown*.

c *Expected and observed haplotypes from a parental breed in Kenyan crossbreds*.

d *Maximum change of haplotype frequency from a parental breed to crossbreds*.

The most frequent haplotypes on BTA 4, 5, 9, 10, 13, and 26 in Holsteins (frequency > 0.3) were shared with Kenyan crossbreds (Table 1), but most these haplotypes were less frequent than expected in crossbreds (Figure 4). Specifically, frequency of the most common haplotypes on BTA 4, 10, 13, 20, and 26 were higher than 0.4 in Holsteins using 50-SNP windows, which were probably inherited from influential ancestors. However, most these haplotypes appear to harbor unfavorable alleles in Kenyan crossbreds since the haplotype from Holstein almost disappeared in crossbreds except for a haplotype on BTA 26. The most frequent haplotypes in the candidate region of crossbreds (Table 1) were found to derive from Holstein-Friesians. As expected, the frequency of the most common haplotypes between Holsteins and Kenyan Friesians was correlated (*r* = 0.55). We observed four noticeable differences (frequency > 0.1) between Holsteins and Kenyan Friesians on BTA 10 (50–60 Mb), 13 (30 Mb), 5 (25, 100 Mb), and 22 (45 Mb). In particular, the intervals on BTA 10 and 13 overlap the regions that encompass the most frequent haplotypes inherited from Holstein ancestors in crossbreds (Table 1).

### Signatures of selection in crossbreds, Holsteins, and Kenyan Friesians

**Figure 5:**
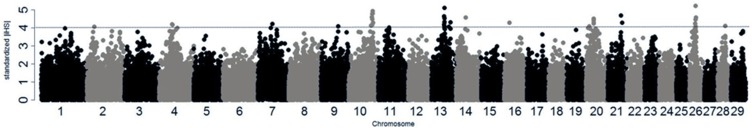
**iHS in Kenyan crossbreds**. |iHS| at each SNP loci is plotted across the genome. Dotted line indicates |iHS| = 4.

We searched for signatures of selection in Kenyan crossbreds, which could support that a given region(s) was probably involved in adaptation. As a consequence, candidate regions can be defined by density of high |iHS| > 3, including the regions on BTA 4, 7, 10, 11, 13, 20, and 26 (Figure 5; Table 2). The signatures of selection on BTA 4, 10, and 20 agreed with those in Kenyan Friesians and Holsteins, whereas the most significant signals on BTA 13 and 26 were detected only in crossbreds. Next, the relatedness of crossbreds and Holstein-Friesians was assessed using Rsb, which may suggest the evidence of common candidate regions in Holsteins and Friesians as well as new candidate regions within crossbreds. However, most significant values of Rsb (Holsteins/Kenyan Friesians) higher than the absolute level of three were positive values, reflecting higher levels of haplotype homozygosity in U.S. Holsteins compared to the EHH of Kenyan Friesians. The F_ST_ between Kenyan crossbreds and Friesians was 0.029, which was lower than the value (0.069) between Holsteins and crossbreds.

**Table 2:** **Signatures of selection in Kenyan crossbreds**.

**BTA**	**Max (iHS)**	**Position (Mb)**	**Region (Mb)^a^**	**Gene^b^**
4	4.21	48.72	37.87–67.08	*MDFIC*
7	4.58	45.01	44.58–49.19	*TRPC6*
10	5.01	87.19	81.45–88.37	*TSHR*
11	4.16	31.43	28.23–32.96	*FBXO11*
13	5.58	47.37	45.07–49.14	*LARP5*
20	4.06	25.5	22.49–39.06	*HSBP3*
26	5.35	23.41	20.10–25.67	*SORCS3*

a *Region defined by at least 3 continuous significant (iHS > 3) per 1 Mb*.

b *Gene located at the loci (or nearest loci) with maximum iHS*.

Additionally, Kenyan Friesians were compared with Holsteins. To assess the similarity between Holstein and Kenyan Friesian cattle, firstly, we measured a differentiation of Holsteins and Friesians using F_ST_. The mean F_ST_ was relatively low (0.012) and only 0.2% of F_ST_ exceeded 0.2 across the genome (Figure S3), which was lower than the mean F_ST_ of Kenyan Friesian and Guernsey (0.078) or Kenyan Friesian and Norwegian Red (0.028). The analysis of EHH revealed several differentially selected regions (|Rsb| > 3) on BTA 1, 3, 5, 9, and 23 that were not found from the results of F_ST_, which may reflect the history recent selection for a few decades compared to F_ST_. However, most regions did not differ substantially across the genome. Then, we carried out the analysis of iHS for each group, resulting in 98 significant signals (|iHS| > 3) in Kenyan Friesians (Figure S4), and only 24 significant loci were detected in Holsteins. Using the iHS, signatures of selection of Kenyan Friesians and Holsteins showed low levels of correlation (*r* = 0.13).

### Genes involved in adaptation

We selected candidate regions that were minimally related to signatures of selection in Holstein-Friesians to find gene(s) that were possibly involved in adaptation. The candidate regions with unique iHS on BTA 11 was chosen first, and then, four regions on BTA 14, 15, and 27 were selected for the excessive ancestry of Holsteins (>0.75). Additionally, the potentially advantageous haplotypes on BTA 16 and 26 in crossbreds were included for this step. The gene *PIK3CD* on BTA 16 is involved in IL-2 signaling and Toll-like receptor (TLR) signaling pathway with *MTOR* (BTA 16) and *NFKB2* (BTA 26), respectively. *IL-2* is a multifunctional cytokine with pleiotropic effects on T cells, B cells, and natural killer cells, and the TLR signaling pathway detects microbial pathogens and is involved in generating innate immune responses. On BTA 11, the genes *LHCGR* and *FSHR* encode receptors of luteinizing hormone/choriogeonadotropin and follicle stimulating hormone that play an important role in the reproductive development process. The gene *CYP17A1* (BTA 26) is also related to the reproductive and gonad development. The candidate region on BTA 14 and 27 was defined at loci with the maximum ratio of Holstein ancestry in flanking region (2 Mb). *NCOA2* (35 Mb) on BTA 14 is related to the regulation of transcription and RNA metabolic process with several genes located on different chromosomal regions, including *RHOQ* (BTA 11), *CTNNBIP1* (BTA 16), *PEX14* (BTA 16), *NFKB2* (BTA 26), *SUFU* (BTA 26), and *LDB1* (BTA 26).

Although haplotypes that originated from Guernsey or Norwegian Reds were not commonly found in Kenyan crossbreds, the analysis of local ancestry revealed the substantial influence of Norwegian Reds in some regions. Haplotypes from Norwegian Red were the most frequent (>0.5) at three regions (>5 Mb) on BTA 5, 20, and 29 (Figure S3) in crossbred cattle, which implies the advantage of Norwegian Red (Ayrshire) background. Among genes located in the regions, *OSMR* on BTA 20 is a notable gene that participates in regulation of inflammatory response, as well as response to cytokine stimulus and cytokine-cytokine receptor interaction with *LIFR* (BTA 20).

## Discussion

In East Africa, there has also been a routine use of Holstein-Friesian semen, whereas the larger footprints of selection in Kenya would be affected by a substantial amount of British Friesian introduction compared to recent Holstein. In Kenya, most of the AI is from the government AI Centre, which uses bulls that have been maintained in the country for many years. A previous study found that the Kenyan admixed cattle population consisted of 30–40% Holstein-Friesian and 60–70% Guernsey (Gorbach et al., 2010), but the crossbreds were not likely to come from only two breeds when one considers more European dairy breeds, including Ayrshire, Guernsey, and Jersey, have been widely used for AI. Furthermore, the ancestry of native African or *bos indicus* are likely to be small in Kenyan dairy cattle.

The Kenyan crossbreds in small farms are expected to be more adapted to local environments, although crossbreeding is not a favorable mating system for dairy cattle in Western countries. Interestingly, we could identify several regions under selection using iHS in Kenyan crossbreds. To interpret this, it is noted that dairy cattle in East Africa have been adapted through crossbreeding of imported breeds and probably some indigenous *bos taurus* cattle that cannot be inferred in this study. The sires used for AI varied in their ancestry with anywhere from 30 to 98% Holstein-Friesians (Gorbach et al., 2010), and the contemporary Friesian herd comprises 45% of the national dairy herd in Kenya. The haplotype based methods tend to be sensitive to the influential common ancestors, implying the effect of an ancestral breed. Thus, we assumed selection signatures in Kenyan crossbreds were greatly dependent on the Holstein genetics. Several candidate regions were detected on BTA 7, 13, 20, and 26 in crossbreds and which overlapped for the same regions with high levels of runs of homozygosity (ROH > 0.16) in the North American Holsteins (Kim et al., 2013). However, we could not clarify the similarity of signature of selection between Kenyan cattle and Western dairy breeds only by comparisons of statistical scores based on the decay of EHH.

Identifying the distinct ancestry of genomic segments has a wide range of applications from disease mapping to learning about history (Sankararaman et al., 2008), and allow us to reveal the favorable local genetic background from a specific breed in an admixed livestock population. The analysis of genomic sequences revealed selection for Asian genes that introgressed into European pigs, demonstrating the effect of artificial selection to improve the fertility in Europe during since nineteenth centuries (Bosse et al., 2014). In Kenyan cattle, migration of haplotypes from Western countries was assessed, while time to the first generation of imported parental breeds may be insufficient to identify adaptation under natural selection. Consequently, adaptation of an allele with low initial frequency is unlikely to be clarified by natural or artificial selection during the last few decades.

Estimates of the most frequent haplotype in crossbreds are fairly similar to half of the corresponding haplotypes in Holstein-Friesians. Norwegian Reds have been selected using a multiple breeding objective, with increasing emphasis on functional traits like health and fertility (Mason, 1988). During the twentieth century, U.S. Holstein and Norwegian Red cattle shared some common ancestors because germplasm of Norwegian Red has been exported to the United States for crossbreeding with Holstein cattle (Heins et al., 2006). PCA also supported the history of this breed. On the contrary, the exact percentage of ancestry is debatable. Among all haplotypes, 17.6% belong to Holstein-Friesian ancestry exclusively, and 1.6 and 0.9% attributed to Norwegian Reds and Guernsey, respectively (Figure S1). Conversely, nearly 80% of haplotypes could belong to any ancestral group. Nonetheless, overall local ancestry information allowed us to reveal possible regions that correspond to adaptation. The excessive ancestry of Holstein (>75%) was inferred at 30 Mb on BTA 14, 80 Mb on BTA15, and 1 Mb on BTA 27 (Figure S4). When comparing to signatures of selection, these regions did not correspond to the selection signatures in Holsteins or crossbred cattle, which implies a potential adaptation of Holstein backgrounds that were not involved in objectives of breeding program in Western countries. Some regions under influence of the same percentage of ancestry, particularly, a region encompassing the MHC from 20 to 30 Mb on BTA 23, may reflect the advantage of high diversity (Figure 4) i.e., high diversity in the MHC derived from diverse ancestors is likely to be beneficial in unfavorable and changing environments.

The ratio of Holstein background could be overestimated in crossbreds due to a limited number of ancestral populations used for HMM. Nevertheless, the measurement of a specific haplotype flow allowed us to infer the remaining ancestry in a genomic region. A comparison revealed that the most frequent haplotype on BTA 26 in crossbreds was found in Holstein-Friesian and Norwegian Red cattle. This region on BTA 26 was also detected using iHS in Kenyan crossbreds, but no sizable signature of selection has been reported in the same region in previous studies of Holstein-Friesians. Clearly, the most common haplotype in this candidate region originated from Holstein-Friesian or Norwegian Red ancestry, which may result in significant standardized score in this region. On a wide region of BTA 26, significant associations of unsaturated fatty acid were detected in Dutch Holsteins (Bouwman et al., 2012), overlapping the candidate region on BTA 26 in Kenyan crossbreds. An obvious change in haplotype frequency was found on BTA 16 where all breeds shared the common haplotype in a very narrow region at 42 Mb. However, the similarity in flanking region was the highest when compared with Kenyan Friesians. Thus, this region may be considered either as resulting from adaptation or differential selection between Holsteins and Friesians.

The results from PCA and F_ST_ supported the assumption that Holsteins did not considerably differ from Friesians. Nevertheless, we could not identify common selection signatures from the results of iHS in Holsteins and Kenyan Friesians. A clue to elucidate common selection signatures was found when surveying haplotypes. Indeed, the pattern of the most frequent haplotype resembles greatly that found in Holsteins and Friesians, except two regions on BTA 10 and 13. The selection signature on BTA 10 was reported in German, Dutch, and U.S. Holsteins (Qanbari et al., 2010; Elferink et al., 2012; Kim et al., 2013), but not in Israeli Friesians (Glick et al., 2012). The frequency of the most common haplotype at 30 Mb on BTA 13 has increased during the last few decades in the U.S. Holsteins, and was associated with milk yield (Kim et al., 2013). Furthermore, one of the largest genomic differences between U.S. Holsteins and Kenyan Friesians were also these two regions on BTA 10 and 13, which may account for the differences between U.S. Holsteins and Kenyan crossbreds. It is also worth mentioning the haplotype migration of BTA 4 from Holsteins to Kenyan crossbreds. On BTA 20, haplotypes inherited from Norwegian Red (Ayrshire) appear to be more frequent than those of Holstein-Friesians in crossbreds. Most previous studies reported strong evidence of selection signatures on BTA 20 in Holstein-Friesians, which agrees with common frequent haplotypes in Holsteins and Friesian in our study. These findings may explain some genetic reasons for the poor production abilities of Kenyan dairy cattle.

We assumed that excessive local ancestry of a specific breed could be an evidence of adaptation. However, in principle, the benefits of crossbreeding are mainly attributed to increased levels of heterozygosity in the F1 generation. Conversely, excessive alleles from only one ancestral breed may not be a desirable inheritance mode in crossbreds unless they confer some advantage. We defined cattle in local farms as Kenyan crossbreds, but their population structure is more complex than crossbreds in Western countries. More importantly, Kenyan crossbreds with imported cattle have been exposed to infectious diseases and other conditions under poor environments. Thus, we emphasized on the contribution of a breed to crossbreds in a local segment of genome using admixture mapping (Shriner, 2013).

Despite existence of common haplotypes, some alleles probably associated with high milk yield were not fully transferred from Holsteins and selected for in crossbreds in local farms. This is not surprising because crossbreeding may pursue higher survivability rather than direct improvement of ability to produce milk yield. However, it is unclear which regions were involved in adaptation to the specific environmental conditions in Kenya without records. One earlier original aim of the Kenyan national AI has been to control the spread of infectious disease among cattle (Duncanson, 1977), but there was no long-term selection program for the resistance to a specific disease. Heat tolerance has not been an objective of dairy breeding in Western countries. Therefore, we regard unique selection signatures or exceptional influence of an ancestral breed in crossbreds as evidence of possible adaptation to East Africa.

For worldwide cattle production in the twenty-first century, it will be necessary to explore the adaptation of cattle in unfavorable environments, where there has been selection for alleles at many loci offering specific environmental adaptation (O'Neil et al., 2010). To overcome low survivability and poor productivity, crossbreeding has been widely applied in East Africa. However, the genomic features underlying the profits of crossbreeding have not been investigated with a view of the entire genome. While three or more breeds are expected to have been used in Kenyan crossbred cattle, the composition of long extended haplotypes has been strongly depended on a few influential bulls that were mostly Holstein-Friesian cattle. Those common haplotypes were probably selected for economic traits rather than survivability in Western countries. Nonetheless, some regions with the excessive ancestry of a specific breed appear to be unrelated to the recent signatures of selection in their parental breed. In the Kenyan crossbreds, Holstein-Friesians background is usually expected to provide the ability of higher milk yield, whereas other breeds may support health or fertility. However, we suggest from the results that some local ancestry of Holstein-Friesians may be advantageous to adaptation to a new environment. Although local adaptation and selection signatures have been identified in Kenyan cattle, these need to be allied to industry efforts to characterize the different aspects of performance in new environments (Rothschild and Plastow, 2014). To better understand adaptation, a genome-wide analysis of local ancestry is required in an admixed population. This type of analysis may enable researchers a clearer view of the details in genetic background that may contribute to survivability. Thus, our results in Kenyan admixed cattle may provide useful information for objective dairy breeding in both temperate and non-temperate climate regions.

### Conflict of interest statement

The authors declare that the research was conducted in the absence of any commercial or financial relationships that could be construed as a potential conflict of interest.
