# Supplementary material for: Genomic adaptation of admixed dairy cattle in East Africa
Source: Front Genet. 2014 Dec 19;5:443. doi: 10.3389/fgene.2014.00443 (PMC4271701; doi:10.3389/fgene.2014.00443)
Supplement: Supplementary file 1 [file SupplementaryFigures.PPTX]

## Slide 1
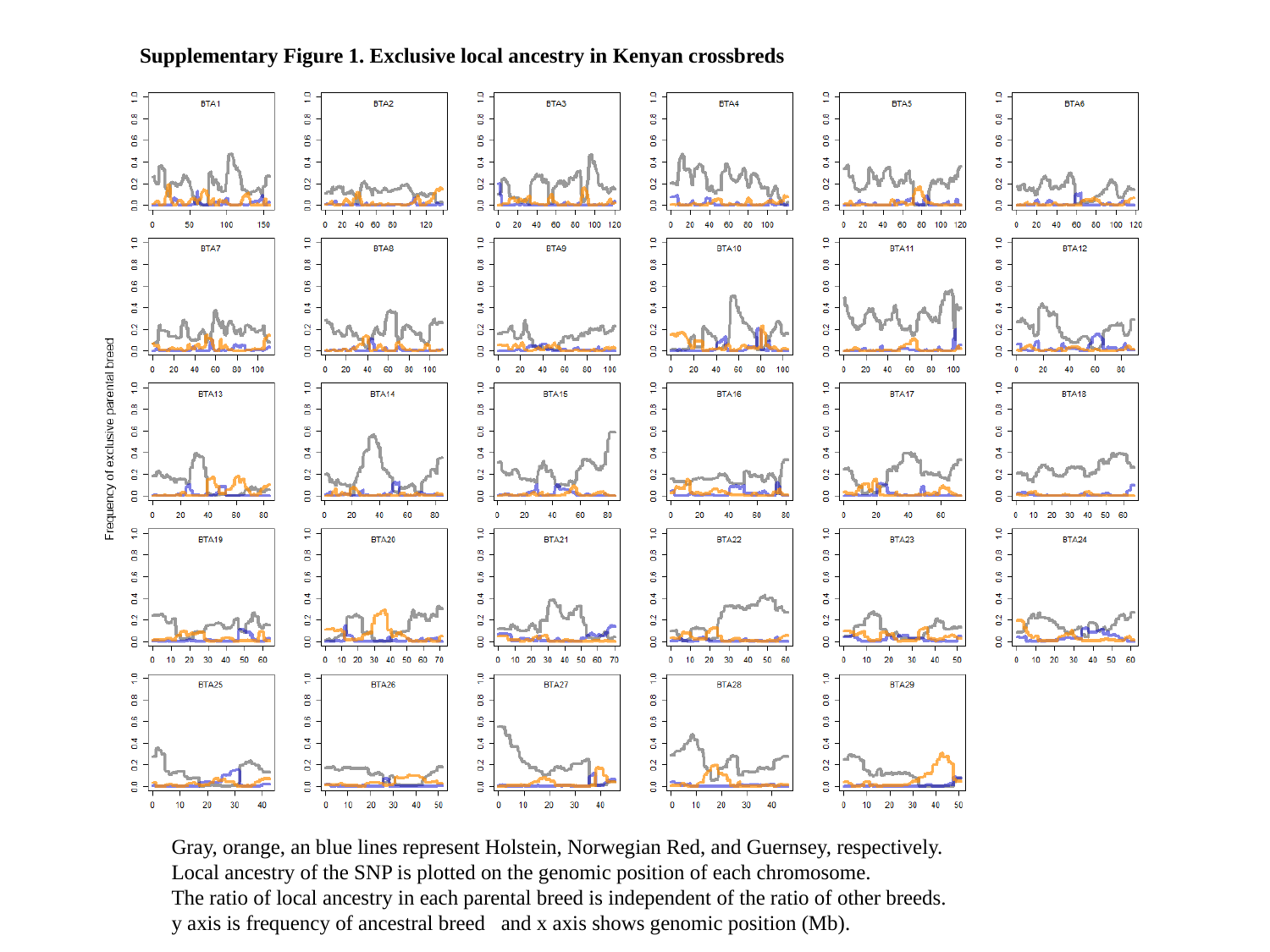

Supplementary Figure 1. Exclusive local ancestry in Kenyan crossbreds
Gray, orange, an blue lines represent Holstein, Norwegian Red, and Guernsey, respectively.
Local ancestry of the SNP is plotted on the genomic position of each chromosome.
The ratio of local ancestry in each parental breed is independent of the ratio of other breeds.
y axis is frequency of ancestral breed and x axis shows genomic position (Mb).

## Slide 2
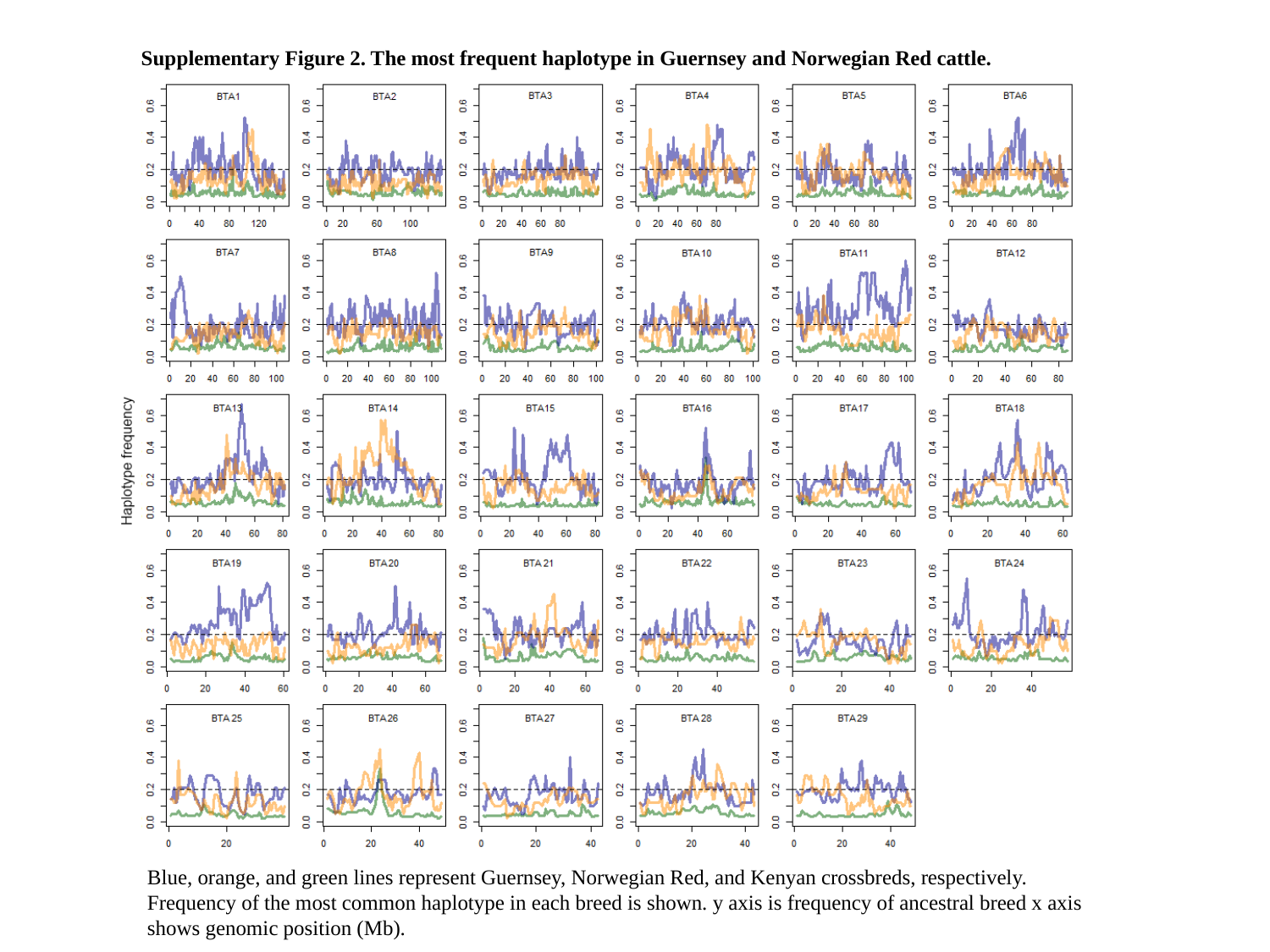

Supplementary Figure 2. The most frequent haplotype in Guernsey and Norwegian Red cattle.
Blue, orange, and green lines represent Guernsey, Norwegian Red, and Kenyan crossbreds, respectively. Frequency of the most common haplotype in each breed is shown. y axis is frequency of ancestral breed x axis shows genomic position (Mb).

## Slide 3
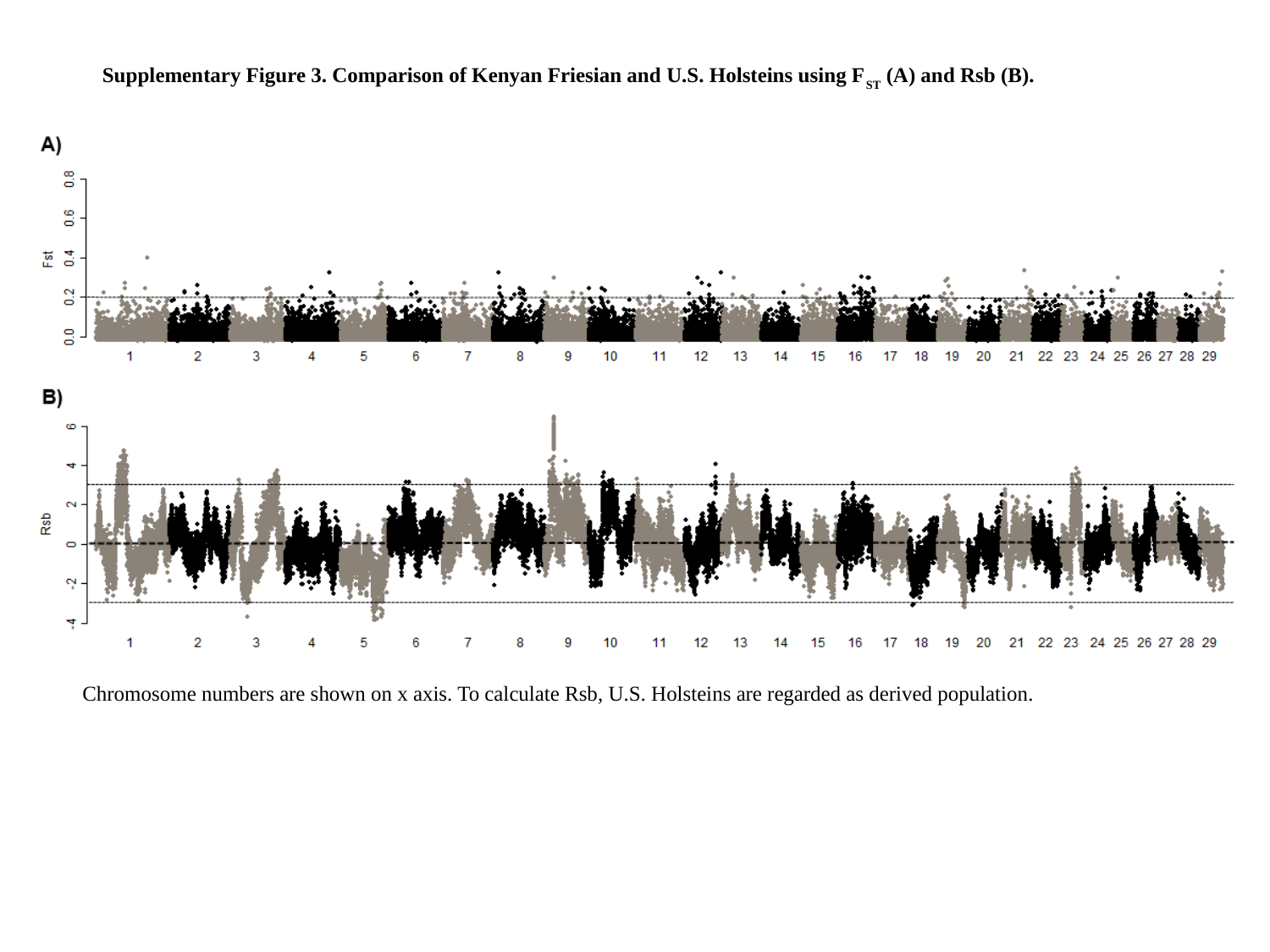

Supplementary Figure 3. Comparison of Kenyan Friesian and U.S. Holsteins using FST (A) and Rsb (B).
Chromosome numbers are shown on x axis. To calculate Rsb, U.S. Holsteins are regarded as derived population.

## Slide 4
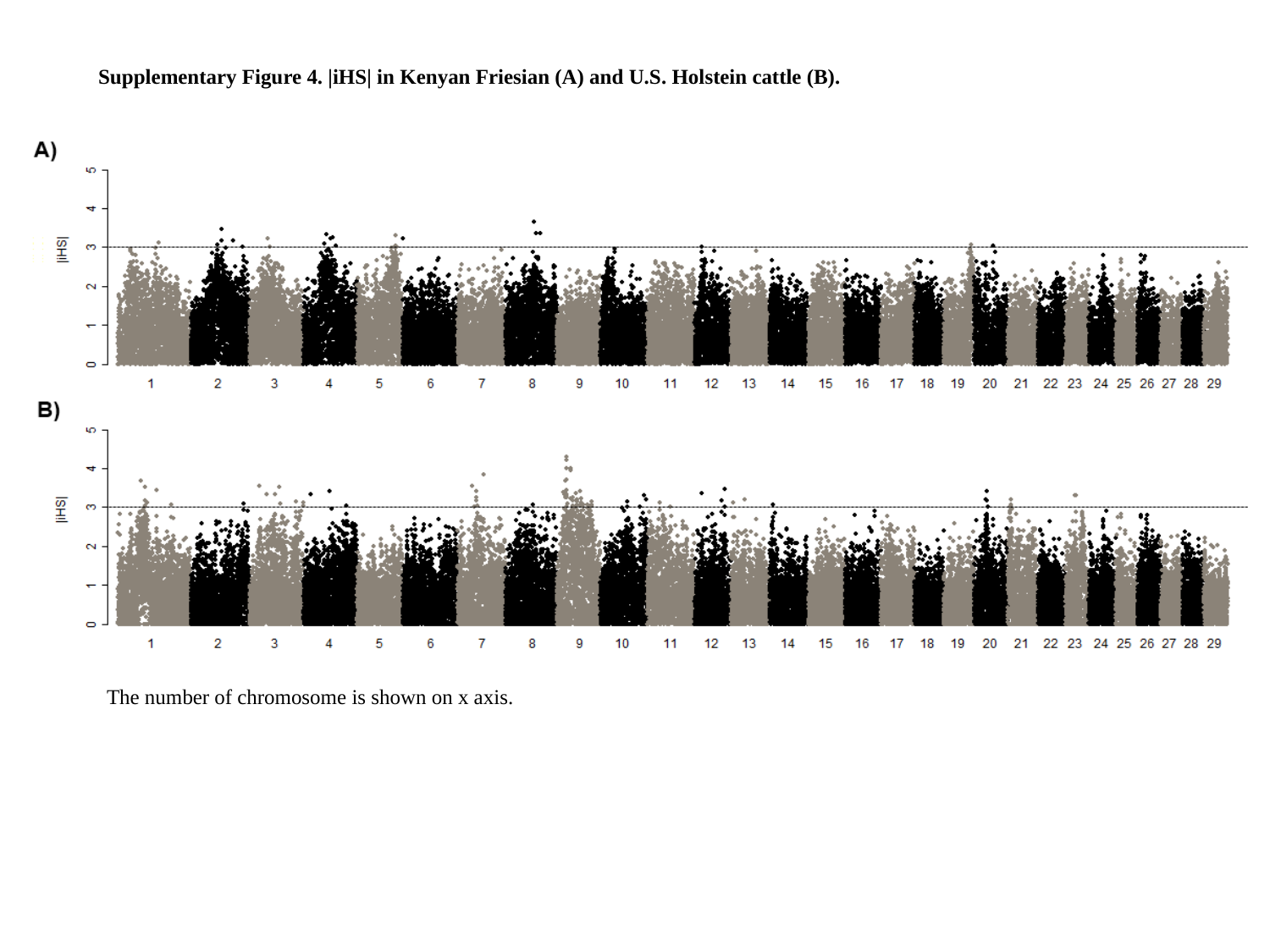

Supplementary Figure 4. |iHS| in Kenyan Friesian (A) and U.S. Holstein cattle (B).
 The number of chromosome is shown on x axis.
